# Association Between Aspirin Usage and Age-Related Macular Degeneration: An Updated Systematic Review and Meta-analysis

**DOI:** 10.3389/fphar.2022.824745

**Published:** 2022-03-25

**Authors:** Ruijia Yan, Jing Zhao, Xinai Zhang, Wei Wang, Zhengyao Jiang

**Affiliations:** Department of Ophthalmology, Qilu Hospital (Qingdao), Cheeloo College of Medicine, Shandong University, Qingdao, China

**Keywords:** long-term use, aspirin, association, age-related macular degeneration, meta-analysis

## Abstract

**Purpose:** To investigate the association between long-term use of aspirin and age-related macular degeneration (AMD).

**Methods:** An updated systematic literature search was conducted in PubMed, Medline, Cochrane Library, and embase from conception to February 26, 2021, without any language restriction. All studies that evaluated the relationship between long-term aspirin use and AMD were included.

**Results:** In the current study, 16 articles were pooled. Overall, no significant association was observed (estimate ratio = 1.108, 95% confidence interval (CI): 0.886–1.385). When the subgroups were evaluated according to various standards, aspirin use was significantly correlated with AMD in studies with volunteer participants (estimate ratio = 0.899, 95% CI: 0.830–0.974, *p* < 0.01), studies followed up for >10 years (estimate ratio = 2.206, 95% CI: 2.124–2.292, *p* < 0.01), duration of aspirin use >10 years (estimate ratio = 2.323, 95% CI: 2.234–2.416, *p* < 0.01), and cohort studies (estimate ratio = 1.961, 95% CI: 1.893–2.032, *p* < 0.01).

**Conclusion:** Therefore, the association of aspirin and AMD can be demonstrated with a long-term follow-up or aspirin use, appropriate study design and participant source. The findings in our study might provide practical information on intervention strategies.

## 1 Introduction

Age-related macular degeneration (AMD) is a progressing disease that mainly affects the macular region of the retina and is the main cause of central vision loss for elderly patients worldwide (Coleman et al., 2008; Lim et al., 2012). The global number of AMD cases is expected to be about 200 million by 2020 (Wong et al., 2014). The clinical signs of AMD were drusen and abnormalities of the retinal pigment epithelium. Age has been identified as a robust risk factor for AMD, with the majority of the AMD patients belonging to the population of >60-year-olds (Joachim et al., 2014; Joachim et al., 2015). The “dry” form of AMD is the most prevalent and advanced form and on the contrary, the “wet” form is responsible for 90% of acute blindness due to AMD (Arruabarrena et al., 2021).

Given the increased retinal vascular permeability and neovascularization associated with AMD, vascular endothelial growth factor (VEGF) inhibition has been considered as one of the effective treatments for AMD (Plyukhova et al., 2020; Dolar-Szczasny et al., 2021). Recently, therapeutic strategies combined with multiple factors, such as daily diet and lifestyle, and preventive pharmacological interventions have been proposed for AMD (Khanani et al., 2022). Aspirin is a widely used antiplatelet drug with proven benefits in lowering cardiovascular risk (Montgomery et al., 2021). A recent European Eye Study reported that regular aspirin usage was associated with AMD, independent of smoking and other risk factors (de Jong et al., 2012). Moreover, aspirin was considered to be associated with macular hemorrhage in AMD cases. A number of multicenter clinical trials have been conducted with regard to this aspect. Although several meta-analyses (Zhu et al., 2013; Kahawita and Casson, 2014; Ye et al., 2014; Li et al., 2015) have assessed the linkage between aspirin usage and AMD, only one meta-analysis reported a weak but statistically significant association (Li et al., 2015).

However, the results on the association between aspirin and AMD were controversial. A previous study conducted by Lee et al. (Lee et al., 2021) reported that patients with regular use of aspirin had a higher risk of developing AMD compared to non-aspirin users. However, another study denied this association (Rim et al., 2019). Nonetheless, another persuasive systematic review and meta-analysis provided specific evidence about this association. In the present study, we systemically updated and evaluated the association of long-term use of aspirin and AMD by summarizing the relevant studies.

## 2 Methods

### 2.1 Literature Search

To obtain relevant studies, several datasets, including PubMed, Medline, Cochrane Library, and embase, were used without language restriction from inception until 26, February 2021. The individual and joint Medical Subject Heading (MeSH) keywords were used to search the literature: “aspirin,” “nonsteroidal anti-inflammatory drugs,” “NSAIDs,” “age-related macular degeneration,” “AMD,” “age-related maculopathy,” and “geographic atrophy.” To include all potentially relevant articles, the bibliography of all relevant studies and reviews were screened for additional eligible studies. Also, Google Scholar was searched for articles that cited related studies. The current study was conducted following the Preferred Reporting Items for Systematic Reviews and Meta-analysis guidelines (PRISMA) statement (Egger et al., 1997).

### 2.2 Eligibility Criteria

The studies were considered eligible if they met the following criteria (Coleman et al., 2008): study population was diagnosed as AMD (Lim et al., 2012); studies that aimed to estimate the association of aspirin use and the risk of AMD (Wong et al., 2014); necessary sufficient data, such as relative risk (RR), odds ratio (OR), hazard ratio (HR) with 95% confidence intervals (CI), or standard error (SE), could be extracted or calculated from original studies (Joachim et al., 2015); studies published in English (Joachim et al., 2014); the study from the same institution providing detailed information or newly published was selected if the population was reported in duplicate.

Case reports, letters, reviews, comments, conference abstracts, studies conducted in animal models or *in vitro* experiments, studies in languages other than English, and studies that were not available were excluded.

### 2.3 Data Extraction

Two reviewers independently searched the above datasets based on the inclusion criteria. The information of the standard-compliant studies was extracted in a standardized form by two reviewers independently, and a consensus on all items was reached by discussion with a third reviewer. For each included study, the following information was extracted: study characteristics (the first author, year of publication, and study design), participant’s characteristics (patient and/or control characteristics, such as mean age and male proportion), diseases characteristics, and results (RR, OR, or HR with 95% CI, or SE).

### 2.4 Quality Scoring of Studies

The quality of the included studies was assessed independently by two authors. Quality assessment and validity tools, Newcastle–Ottawa scale (NOS) and the Jadad scale, were used to assess the methodological quality of observational studies (Wells et al., 2000) and randomized clinical trials (RCTs) (Jadad et al., 1996), respectively.

The NOS for grading observational studies was based on three factors: selection of participants, comparability of each group, and exposure of factors. The score ranged from two to nine points. A scale of 0–2 points indicated poor quality, three to five points as a medium, and six to nine points as high quality. To explore potential heterogeneity, studies with low or medium quality were used for sensitivity analysis.

The overall Jadad score ranged from 0 to 5. For setting a minimum standard for the results to be included in the meta-analysis, studies with Jadad score <3 was defined as low quality and excluded (Ning et al., 2013).

### 2.5 Statistical Analysis

RR or for each included study was pooled, and the corresponding 95% confidence intervals (CIs) were calculated. The inverse variance methods with random-effects model were used to pool the results of the included studies. The subgroup analyses were carried out according to study country, participant source, aspirin dosage, AMD stage, follow-up time and study design. The standard heterogeneity test based on the *I* ^*2*^ statistic was used to assess the consistency of the effect sizes. The heterogeneity was categorized into with and without according to the values of *I* ^*2*^ ≥ 50% and <50% (Higgins et al., 2003), respectively. To explore the sources of heterogeneity with *I* ^*2*^ ≥ 50% (significant heterogeneity), all enrolled studies were sequentially excluded from demonstrating the overall effect. The publication bias was assessed by Begg’s rank correlation (Begg and Mazumdar, 1994) and Egger’s weighted regression methods (Egger et al., 1997). The duplicated study population in the study ws excluded and estimated the overall association between aspirin use and AMD. The forest plot was constructed, and statistical analyses were performed using RevMan version 5.3. Statistical analyses and Begg’s and Egger’s tests were conducted using STATA 15.0 (Stata Corporation, College Station, TX, United States). *p*-value of <0.05 indicated statistical significance.

## 3 Results

### 3.1 Study Selection

**FIGURE 1:**
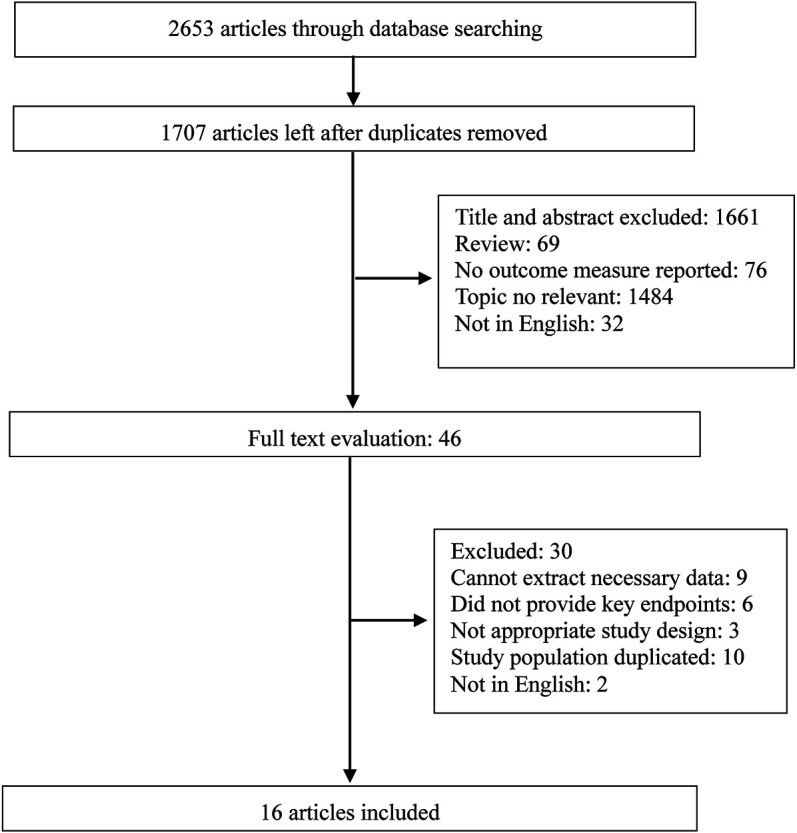
Flowchart of the study selection.

The study selection flowchart is illustrated in Figure 1. The systematic literature search yielded 2,653 studies by the search strategy, and 946 were excluded due to duplication. Based on the above inclusion and exclusion criteria, 1,661 abstracts and titles were reviewed initially. After retrieving 46 full-length manuscripts, 16 articles (Blumenkranz et al., 1986; Christen et al., 2001; Klein et al., 2001; DeAngelis et al., 2004; Clemons et al., 2005; Douglas et al., 2007; Christen et al., 2009; Rudnicka et al., 2010; de Jong et al., 2012; Klein et al., 2012; Cheung et al., 2013; Liew et al., 2013; Modjtahedi et al., 2018; Keenan et al., 2019; Rim et al., 2019; Lee et al., 2021) were included for data extraction and meta-analysis.

### 3.2 Study Characteristics

Among the 16 included studies, a total of 1002092 AMD participants were included. The included studies were published between 1986 and 2021. The sample size ranged from 49 to 682133 AMD Patients. The eight included studies were conducted in the United States (Blumenkranz et al., 1986; Christen et al., 2001; Klein et al., 2001; DeAngelis et al., 2004; Clemons et al., 2005; Christen et al., 2009; Klein et al., 2012; Modjtahedi et al., 2018), one each in UK (Rudnicka et al., 2010), China (Lee et al., 2021), Singapore (Cheung et al., 2013), South Korea (Rim et al., 2019), and Australia (Liew et al., 2013), and one each in seven European countries (de Jong et al., 2012) including Bergen (Norway), Tallinn (Estonia), Belfast (UK), Paris-Creteil (France), Verona (Italy), Thessaloniki (Greece), and Alicante (Spain). Among the studies, five were case-control, two were RCTs, six were cohorts, and three were cross-sectional studies. The various stages of cases, including early-, late-, any-stage, neovascular geographic atrophy AMD, and AMD with or without vision loss, were assessed. Moreover, the majority of the studies were population- or volunteer-based studies. The characteristics of the study population are presented in Table 1.

**TABLE 1:** Characteristics of study participants.

Studies included	Country	Study design	Population source	Aspirin dose	Duration of aspirin use (years)	Median follow‐up (years)	AMD classification	Sample size
Blumenkranz et al., 1986	United States	Case-control	Clinic based	Ever or never used	NA	NA	Neovascular Geographic atrophy	49
Klein et al., 2001	United States	Case-control	Population-based	Not mentioned	NA	5	Early stage	3,684
Christen et al., 2001	United States	RCT	Volunteer-based (males)	325 mg/2 days	>5 years	5	With or without vision loss	21216
DeAngelis et al., 2004	United States	Case-control	Clinic based	>2 times/week	>6 months	NA	Neovascular Geographic atrophy	146
Clemons et al., 2005	United States	Cohort	Clinic based	Ever or never used	>5 years	6.3	Neovascular Geographic atrophy	4,757
Douglas et al., 2007	UK	Case-control	Population-based	Ever or never used	NA	NA	Any stage	104,176
Christen et al., 2009	United States	RCT	Volunteer-based (females)	100 mg/2 days	>10 ears	10	With or without vision loss	39876
Rudnika et al., 2010	UK	Case-control	Clinic based	Ever or never used	NA	3	Late stage	158
deJong PT et al., 2012	European^a^	Cross sectional	Population-based	Range from neverto daily use	NA	NA	Early and late AMD	4,691
Klein et al., 2012	United States	Cohort	Population-based	>2 times/week	>5 years	14 8	Early stage	4,926
Cheung et al., 2013	Singapore	Cross sectional	Population-based	Ever or never used	NA	NA	Early stage	3,207
Liew et al., 2013	Australia	Cross sectional	Population-based	Regular user	>1 month	15	Any stage	2,389
Modjtahedi et al., 2018	United States	Cohort	Volunteer-based (males)	Ever or never used	New/4 years/NA	7.4	Any stage	51731
Rim et al., 2018	South Korea	Cohort	Population-based	Long-term regular use of low-dose aspirin	>5 years	5	Any stage	74196
Keenan et al., 2019	United States	Cohort	Clinic based	Ever or never used	>10 years	10.1	Neovascular Geographic atrophy	4,757
Lee et al., 2021	China	Cohort	Population-based	2 times/week	>10 years	10	Any stage	682,133

Abbreviations: AMD, Age-related macular degeneration; RCT, Randomized Controlled Trial; NA, not available.

a 7 European countries: Bergen (Norway), Tallinn (Estonia), Belfast (United Kingdom), Paris—Creteil (France), Verona (Italy), Thessaloniki (Greece), and Alicante (Spain).

### 3.3 Quality Assessment of Studies

According to the scale of the published quality assessment and validity tool for correlational studies, none of the studies were assessed as low quality (NOS <6 or Jadad score <3). The detailed scores for each included study are shown in Supplementary Table S1.

### 3.4 Overall Association Between Aspirin Use and AMD

**FIGURE 2:**
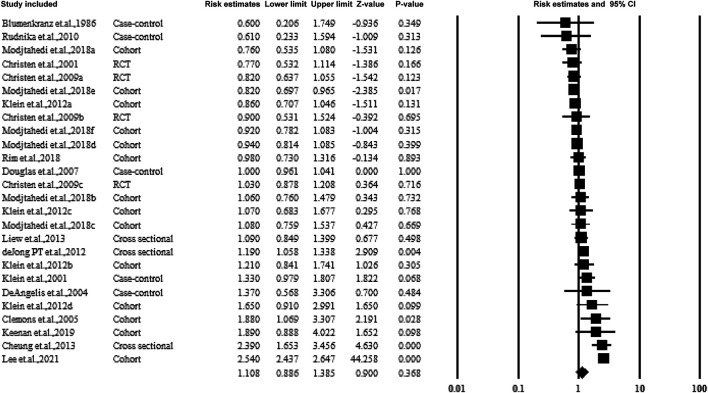
Summarized overall estimate ratio.

Figure 2 shows the overall estimate ratio of the association between aspirin use and AMD. A total of 16 studies reported the overall oral microbiome in pancreatic cancer cases. The individual estimate ratio ranged from 0.600 to 2.540. When the estimated ratio was pooled, no significant association was observed with the overall estimate ratio and 95% CI being 1.108 (0.886–1.385) without heterogeneity (*I* ^*2*^ = 21%).

### 3.5 Subgroup Analysis for the Association Between Aspirin Use and AMD

#### 3.5.1 Subgroup Analysis by Study Country

**FIGURE 3:**
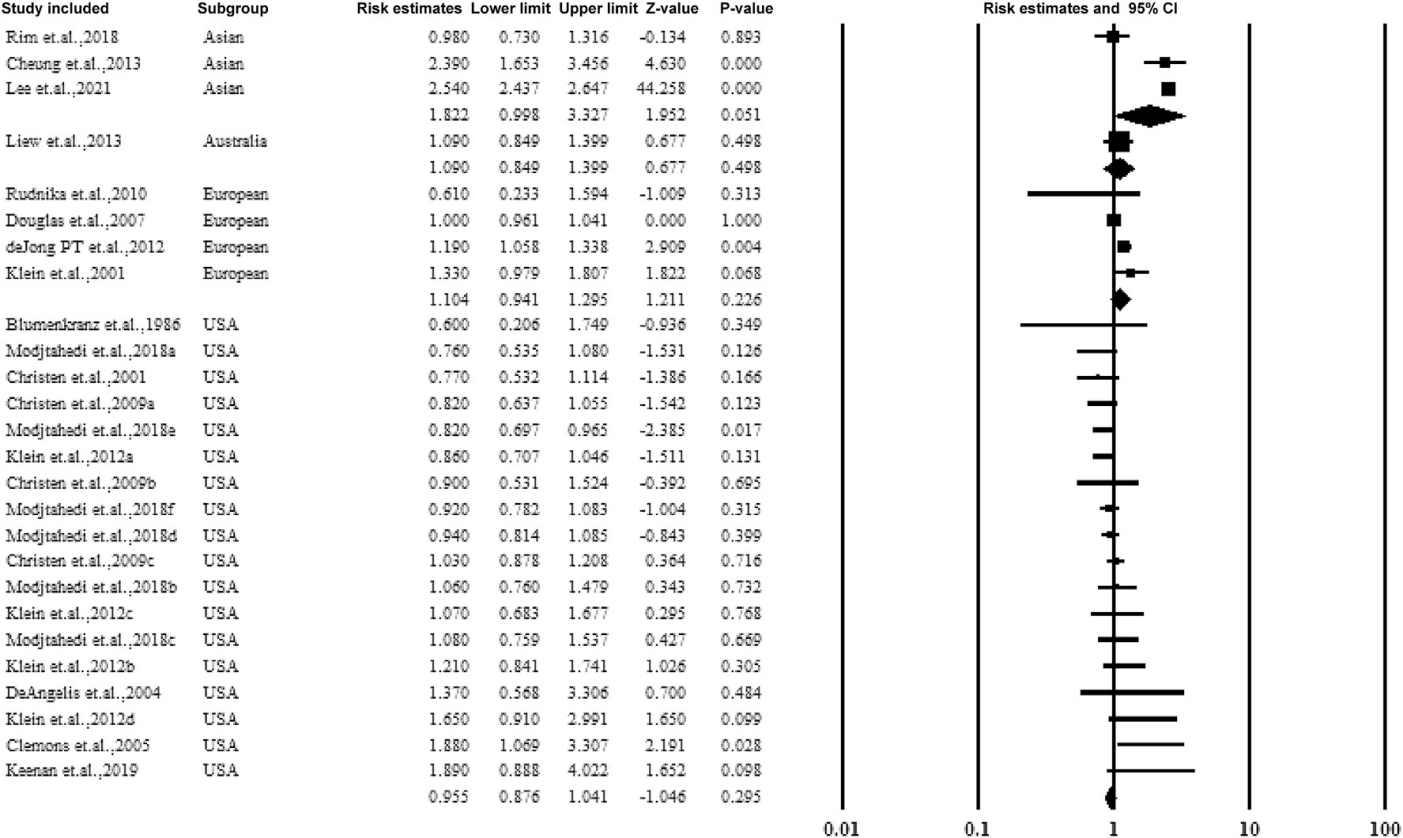
Summarized subgroup analysis according to the study countries.

Figure 3 shows the data on subgroups to explore the effect of the study conducted in various countries. Four categories included the United States, Asia, Australia, and European countries. When pooling the results together, no significant association was established between the overall estimate ratio and 95% CI. The values were 0.955 (0.876–1.041), 1.8922 (0.998–3.327), and 1.104 (0.941–1.295) for United States, Asia, and European countries, respectively.

#### 3.5.2 Subgroup Analysis Based on the Source of the Participants

**FIGURE 4:**
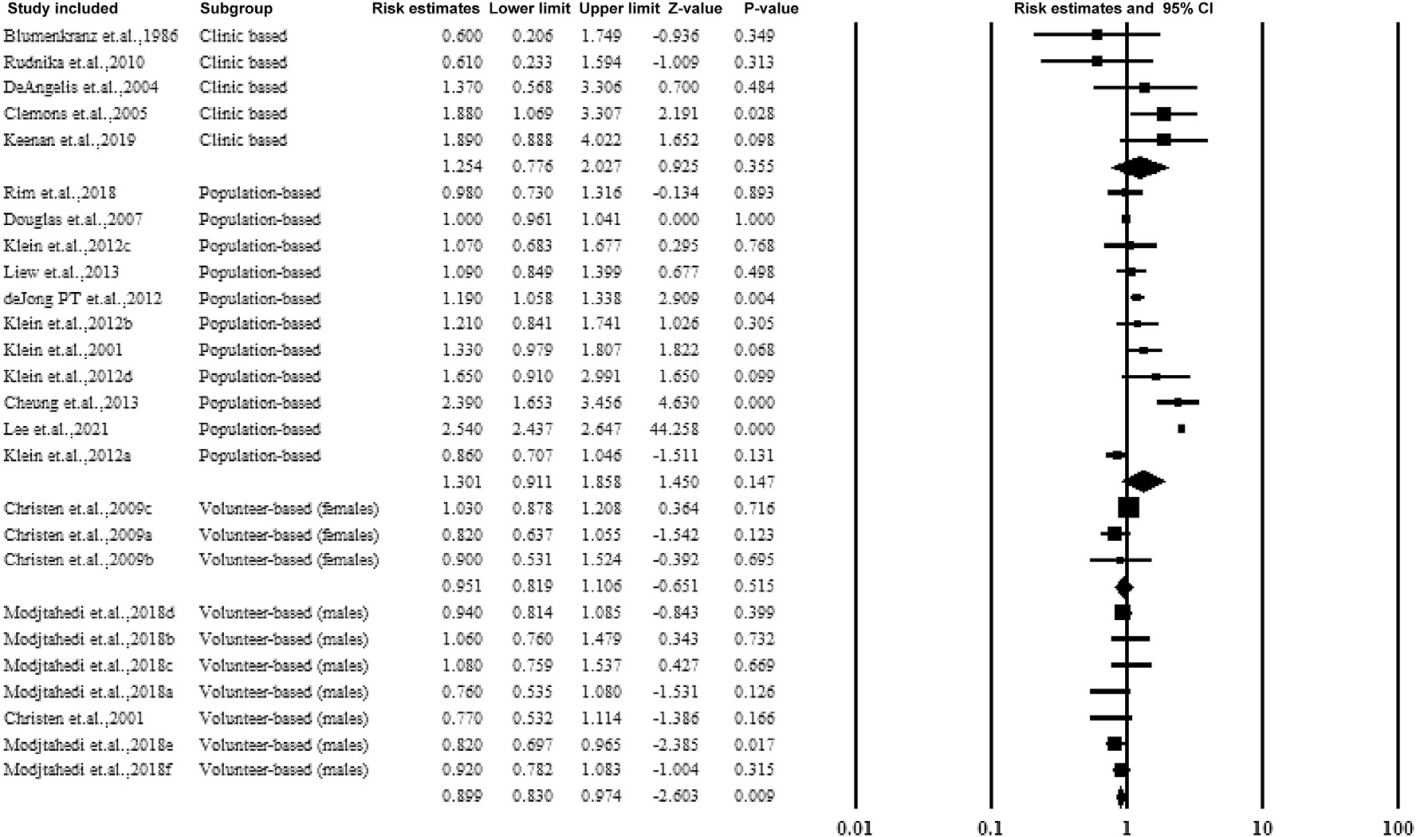
Summarized subgroup analysis according to the source of the participants.

The studies were categorized according to the source of participants, including clinical-based, population-based, and volunteer-based studies. Two studies included only male volunteers and assessed the association of aspirin use and AMD with an estimated ratio of 0.899 (95% CI: 0.830–0.974, *p* < 0.01, *I* ^*2*^ = 0%). The forest plot is shown in Figure 4.

#### 3.5.3 Subgroup Analysis by Dose or the Frequency of Taking Aspirin

**FIGURE 5:**
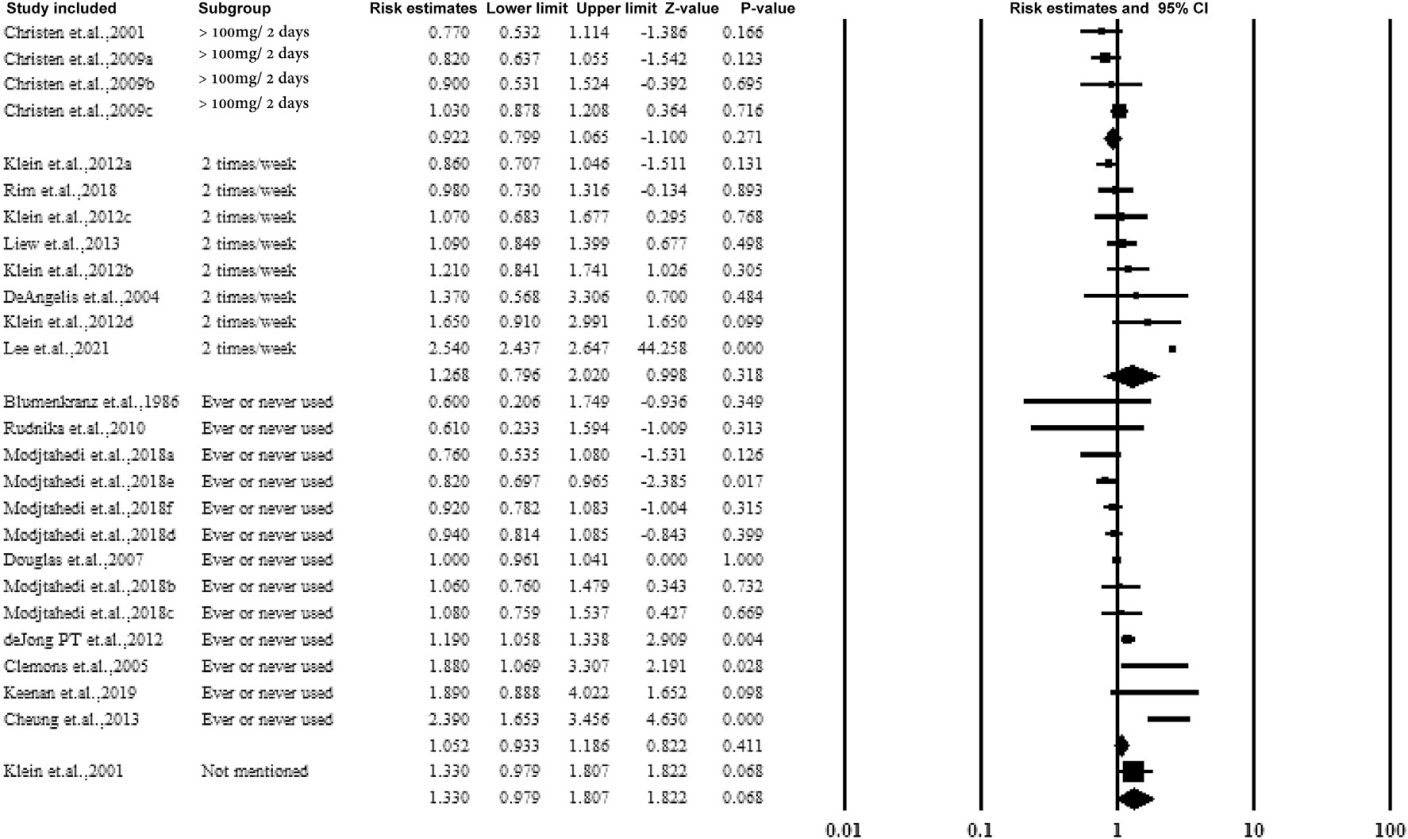
Summarized subgroup analysis according to the dose or frequency of taking aspirin.

The majority of the studies collected the dose or frequency of taking aspirin by questionnaire. According to the dose or frequency, aspirin was categorized into >100 mg/2 days or 2 times/week as regular ingestion. However, no association was observed, and the data are presented in Figure 5.

#### 3.5.4 Subgroup Analysis by the Stage or Classification of AMD

**FIGURE 6:**
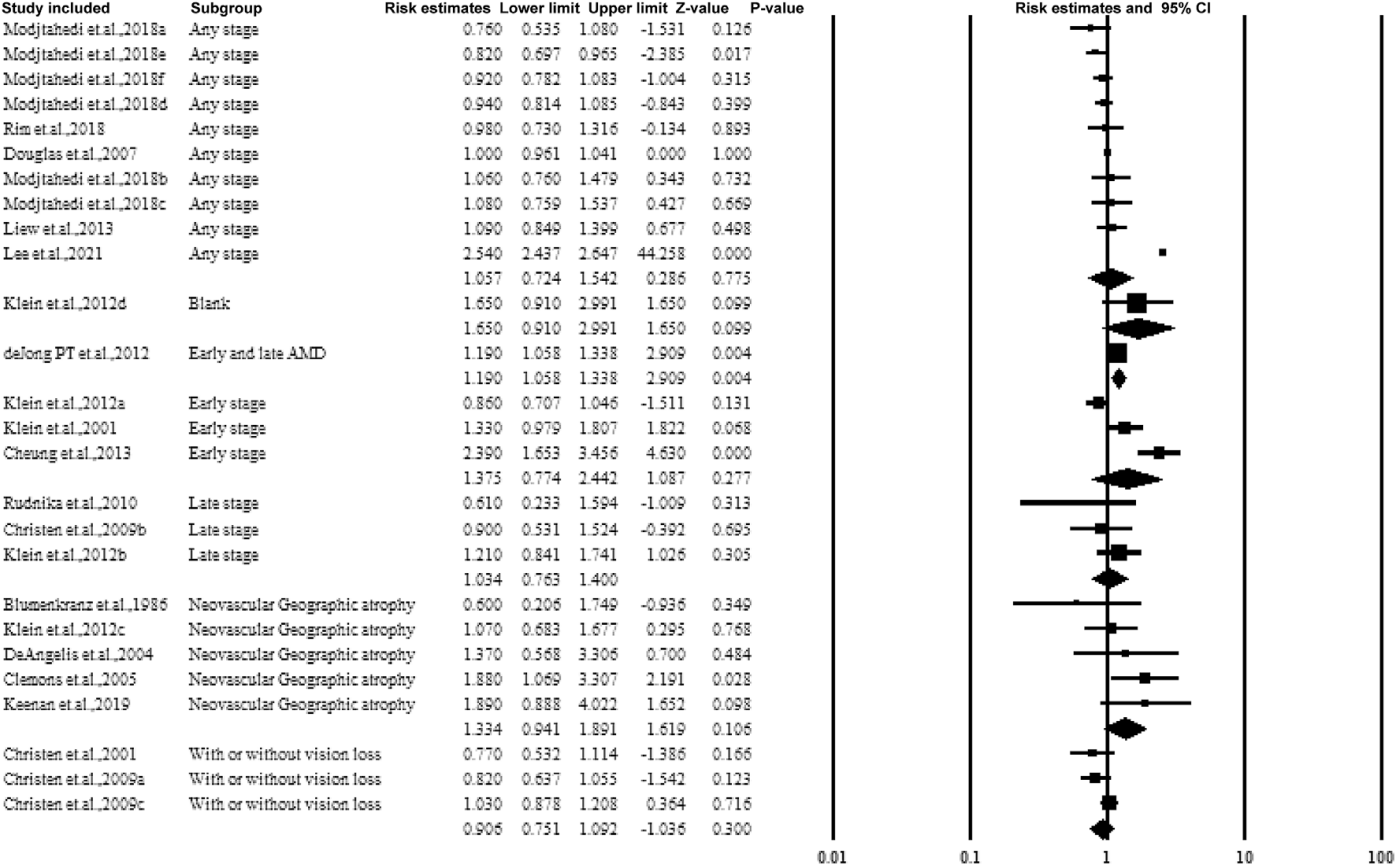
Summarized subgroup analysis according to the stage or classification of AMD.

Figure 6 shows the forest plot of subgroup analysis according to the stage of AMD. Four groups including early, late, any stage, neovascular geographic atrophy, and with or without vision loss. While pooling the results, similar to categorizing by dose or frequency of taking aspirin, no association was observed.

#### 3.5.5 Subgroup Analysis by Follow-Up Years and Duration of Aspirin Use

**FIGURE 7:**
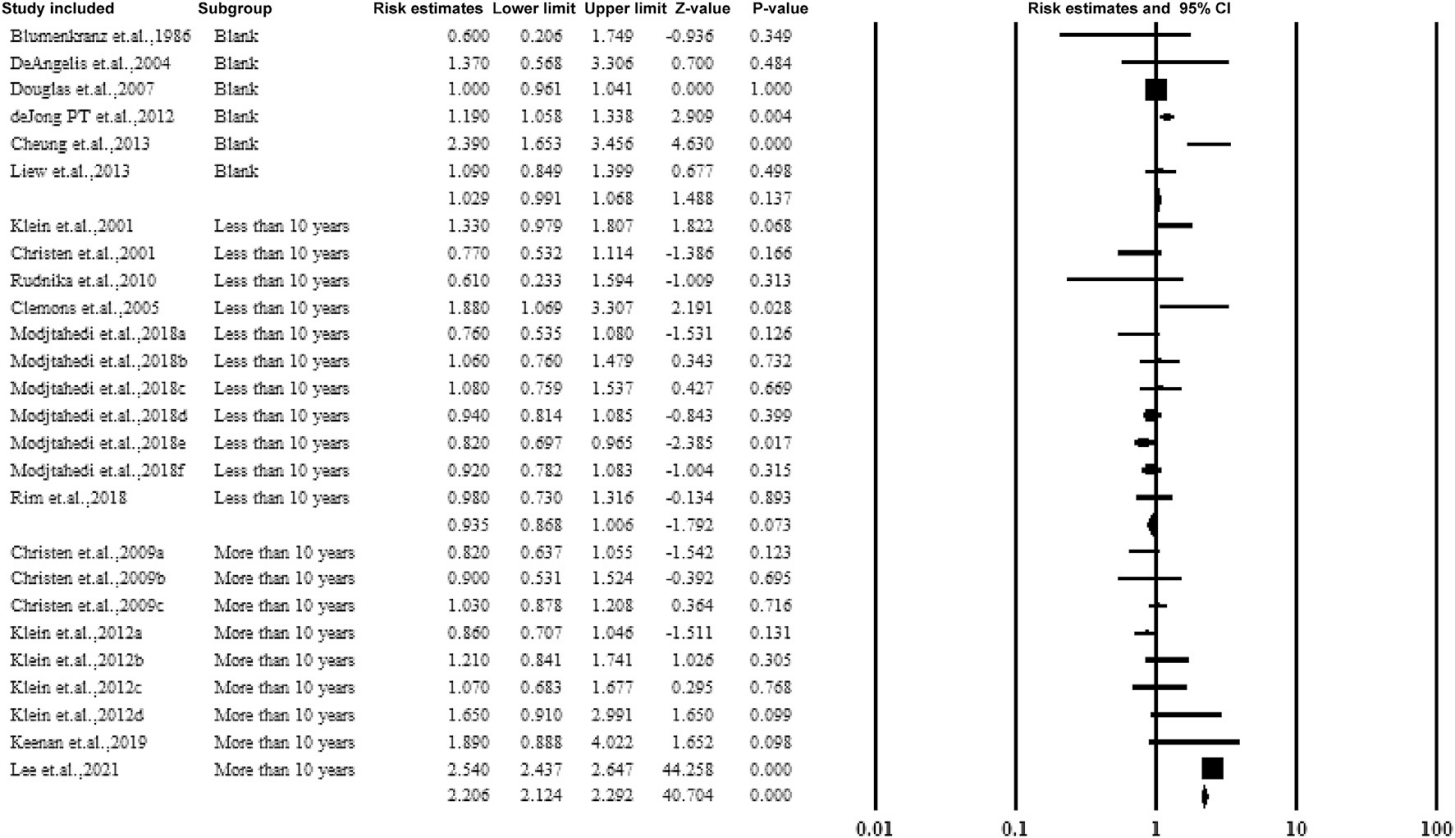
Summarized subgroup analysis according to the follow-up years.

For cohorts and case-control studies, the participants were followed up several times. The studies were classified according to the follow-up durations: <10 years, >10 years, and without follow-up. The studies followed up for >10 years showed a correlation between aspirin use and AMD with an estimated ratio of 2.206 (95% CI: 2.124–2.292, *p* < 0.01, *I* ^*2*^ = 0%) (Figure 7).

**FIGURE 8:**
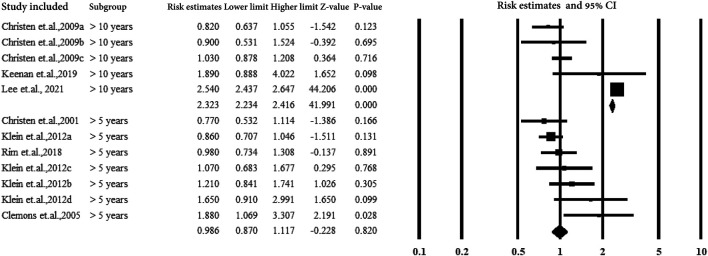
Summarized subgroup analysis according to the study design.

Figure 8 shows subgroup analysis by duration of aspirin use (>5 years and >10 years). The subjects with aspirin use >10 years showed a strong correlation between aspirin use and AMD with an estimated ratio of 2.323 (95% CI: 2.234–2.416, *p* < 0.01, *I* ^*2*^ = 0%) (Figure 7).

### 3.5.6 Subgroup Analysis by Study Design

**FIGURE 9:**
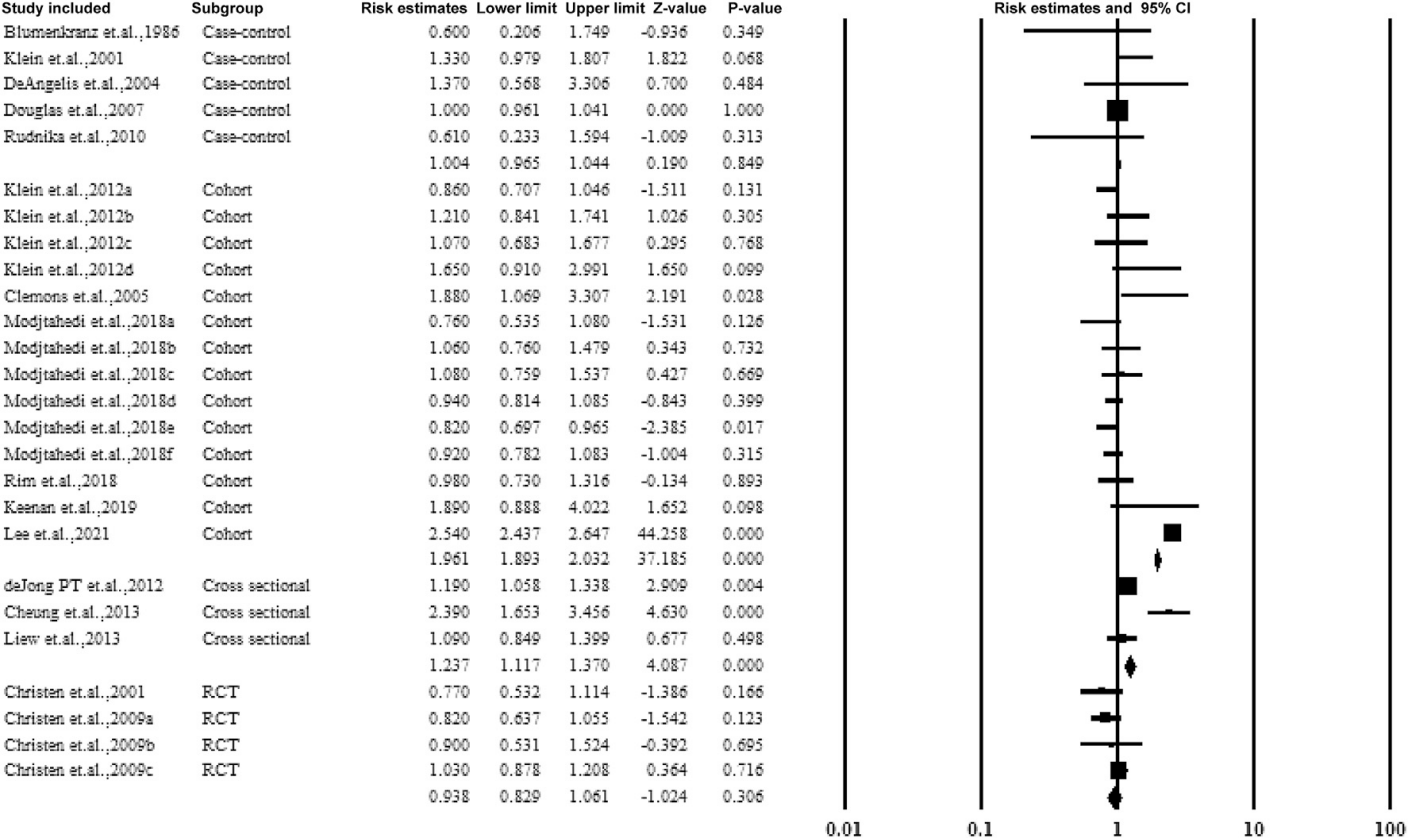
Christen et al., 2009a–c: data for visually significant AMD, advanced AMD, and AMD with or without vision loss, respectively. Modjtahedi et al., 2018a–f, data for longer use-, new user-, and former-user of aspirin on exudative AMD, longer use-, new user-, and former-user of aspirin on nonexudative AMD, respectively.

Figure 9 presents the subgroup analysis of the association between aspirin and AMD according to the study design. The four study types included RCT, cohort, case-control, and cross-sectional studies. The cohort studies observed an association between aspirin and AMD (estimated ratio = 1.961, 95% CI: 1.893–2.032, *p* < 0.01).

### 3.6 Publication Bias

No potential publication bias was detected among the included studies, according to Begg’s rank correlation analysis and Egger’s weighted regression analysis (all *p*-values > 0.05, Supplementary Table S2).

## 4 Discussion

In the current study, 16 studies were summarized and pooled. The overall estimate ratio of the association between aspirin and AMD was observed without statistical significance. The subgroups were evaluated according to various standards, the study participants were volunteers, the studies were followed-up for>10 years or aspirin use >10 years, and cohort studies demonstrated the correlation of aspirin with AMD.

Since 1986, various studies have attempted to demonstrate the association of aspirin use and AMD (Blumenkranz et al., 1986). Several studies have been conducted to establish the association of aspirin with the occurrence and progression of AMD, but the results were inconsistent. Four meta-analyses (Zhu et al., 2013; Kahawita and Casson, 2014; Ye et al., 2014; Li et al., 2015) were published. The study conducted by Ye et al. included 10 studies and suggested that aspirin use was not associated with AMD (Ye et al., 2014). Another study was conducted on AMD by Kahawita et al. (Kahawita and Casson, 2014) in 2013, which summarized four studies. A small but statistically significant association was observed between aspirin use and early ARMD. The meta-analysis conducted by Li et al. (Li et al., 2015) also reported a similar result. However, another meta-analysis denied the association based on the results pooled from the other ten studies. Aspirin is one of the most widely used medicine acutely or chronically in millions of people for differently reducing pain and fever, treating inflammatory diseases, or preventing cardiovascular diseases (Klein et al., 2012; Cheung et al., 2013). Moreover, aspirin produces an array of undesired effects, including dyspepsia and gastrointestinal problems (Klein et al., 2012; Cheung et al., 2013). AMD is a disease that develops slowly and insidiously, with clinical symptoms among the elderly population, and the pathogenesis of AMD is complex and unclear. The anti-inflammatory actions of aspirin may play a role in slowing the process of vision loss via a low-grade inflammatory process. However, the correlation between aspirin use and AMD is not yet proven. Aspirin is an anti-inflammatory agent that prevents inflammation and decreases related damage. In addition, aspirin was shown to affect the COX-independent pathway, and Wnt and HO-1 pathways were reported to be related to AMD (Zhou et al., 2010). A putative linkage was shown between aspirin and AMD. Moreover, aspirin might also cause harmful effects on AMD. A recent study reported that a NSAID, including aspirin, blocked the gap junction communication between the RPE cells and the damaged retinal microenvironment, which promoted the progression of AMD (Ning et al., 2013). It has not yet explored to reduce and prevent AMD by balancing the risks and benefits of aspirin use. The current review aimed to resolve the correlation described above.

In our study, we found that aspirin was associated with AMD in studies having a long-term follow-up (>10 years) or cohort design. A possible explanation is that the aspirin has a weakness effect on AMD and only a long term and accumulative factor can lead to a significant harmful effect. The published studies reported inconsist results on that. The study conducted by Klein et al. (Klein et al., 2012) reported that regular aspirin use 10 years was associated with a weaken increased risk of AMD. Another randomized trial of women reported no harmful effect of aspirin use on risk of AMD (Christen et al., 2009). During a long term aspirin use, the correlation might be effected by various potential existing confounding factors and the study design. The modest contribution of aspirin use on the risk of AMD therefore required continued validation by additional epidemiological researches and in high-quality clinical trials, particularly in random control trials conducted in large scale and whole-age population.

The main strength of the current meta-analysis is systemical search of relevant studies, including a large number of studies, and multiple subgroup analyses. Nevertheless, the present meta-analysis had some limitations while interpreting the results. First, most were observational studies, which might limit the ability to estimate causality and decrease the generalizability of the results. Second, the number of the included studies varied largely, and the majority of the studies were conducted in Western countries and focused on the Caucasian population. The current results might be affected by environmental, medical, and genetic factors, and the representativeness of the target population wasweakened. Third, the dose and frequency of aspirin were based on questionnaires. These features could cause recall bias. Fourth, in the subgroup analysis, we included more than one studies that obtained from one paper. That might also lead to potential bias, i.e. the pooled results might highly rely on these studies and might affected by the characteristics of these studies. Fifth, language bias might be detected because our literature search only considered the articles published in English.

In conclusion, in the current meta-analysis, we systematically assessed the correlations between aspirin and AMD, and the pooled results were based on 16 studies from seven different regions or countries. Also, no statistical significance was detected in the overall association between aspirin and AMD. However, for subgroups analysis, the studies consisting volunteer participants, studies followed up or duration of aspirin use more than 10 years, and cohort studies suggested the association between aspirin and AMD. These fingings in our study might provide useful information in formulating intervention strategies. Nonetheless, these results need to be verified.

## Data Availability

The original contributions presented in the study are included in the article/Supplementary Material, further inquiries can be directed to the corresponding author.
