# Supplementary material for: Association Between Aspirin Usage and Age-Related Macular Degeneration: An Updated Systematic Review and Meta-analysis
Source: Front Pharmacol. 2022 Mar 25;13:824745. doi: 10.3389/fphar.2022.824745 (PMC8990128; doi:10.3389/fphar.2022.824745)
Supplement: Supplementary file 1 [file Table1.DOCX]

**Supplementary Table 1. Quality assessment of included studies by Newcastle-Ottawa Scale ^a^ or Jadad score ^b^**

| **Study included** | **Score name** | **Overall quality score** |
| --- | --- | --- |
| Blumenkranz et.al.,1986 | NOS | 7 |
| Klein et.al.,2001 | NOS | 7 |
| Christen et.al.,2001 | Jadad | 4 |
| DeAngelis et.al.,2004 | NOS | 7 |
| Clemons et.al.,2005 | NOS | 6 |
| Douglas et.al.,2007 | NOS | 8 |
| Christen et.al.,2009 | Jadad | 4 |
| Rudnika et.al.,2010 | NOS | 6 |
| deJong PT et.al.,2012 | NOS | 7 |
| Klein et.al.,2012 | NOS | 7 |
| Cheung et.al.,2013 | NOS | 7 |
| Liew et.al.,2013 | NOS | 8 |
| Modjtahedi et.al.,2018 | NOS | 6 |
| Rim et.al.,2018 | NOS | 6 |
| Keenan et.al.,2019 | NOS | 7 |
| Lee et.al.,2021 | NOS | 7 |

^a^, The study quality was assessed according to the Newcastle Ottawa Quality assessment scale for cross-sectional studies or cohort studies. This scale awards a maximum of 9 points to each study.

^b^, 1 point if randomization is mentioned, 1 additional point if the method of randomization is appropriate. Deduct 1 point if the method of randomization is inappropriate (minimum 0); 1 point if blinding is mentioned, 1 additional point if the method of blinding is appropriate. Deduct 1 point if the method of blinding is inappropriate (minimum 0); the fate of all patients in the trial is known. If there are no data, the reason is stated.
