# Supplementary material for: Association Between Aspirin Usage and Age-Related Macular Degeneration: An Updated Systematic Review and Meta-analysis
Source: Front Pharmacol. 2022 Mar 25;13:824745. doi: 10.3389/fphar.2022.824745 (PMC8990128; doi:10.3389/fphar.2022.824745)
Supplement: Supplementary file 2 [file Table2.DOCX]

**Supplementary Table 2. Publication bias of summarized outcomes**

| **Outcomes** | **Begg (*P*-value)** | **Egger (*P*-value)** |
| --- | --- | --- |
| Summarized overall estimate ratios | 0.85 | 0.47 |
| Summarized subgroup analysis of Asian | 0.17 | 0.56 |
| Summarized subgroup analysis of European | 0.66 | 0.15 |
| Summarized subgroup analysis of USA | 0.19 | 0.14 |
| Summarized subgroup analysis of clinical based | 0.07 | 0.10 |
| Summarized subgroup analysis of population based | 0.13 | 0.09 |
| Summarized subgroup analysis of volunteer based | 0.19 | 0.17 |
| Summarized subgroup analysis of >100 mg/ 2 days | 0.34 | 0.17 |
| Summarized subgroup analysis of 2 times/ week | 0.55 | 0.74 |
| Summarized subgroup analysis of ever or never used | 0.09 | 0.19 |
| Summarized subgroup analysis of any stage | 0.65 | 0.23 |
| Summarized subgroup analysis of early stage | 0.41 | 0.32 |
| Summarized subgroup analysis of late stage | 0.36 | 0.21 |
| Summarized subgroup analysis of NGA | 0.98 | 0.14 |
| Summarized subgroup analysis of with or without vision loss | 0.74 | 0.54 |
| Summarized subgroup analysis of without follow up | 0.15 | 0.10 |
| Summarized subgroup analysis of follow-up less 10 years | 0.74 | 0.70 |
| Summarized subgroup analysis of follow-up more 10 years | 0.66 | 0.47 |
| Summarized subgroup analysis of duration of aspirin use > 5 years | 0.54 | 0.69 |
| Summarized subgroup analysis of duration of aspirin use > 10 years | 0.21 | 0.11 |
| Summarized subgroup analysis of case-control study | 0.47 | 0.32 |
| Summarized subgroup analysis of cohort | 0.51 | 0.09 |
| Summarized subgroup analysis of cross-sectional study | 0.14 | 0.31 |
| Summarized subgroup analysis of RCT | 0.09 | 0.15 |
